# How much is it worth a smile?

**DOI:** 10.1590/2176-9451.20.3.011-012.edt

**Published:** 2015

**Authors:** David Normando

Studying and striving for truth and beauty in general is a sphere in which we are allowed to be children throughout life. **Albert Einstein (physicist)**

Not long ago, about 400 orthodontists attended an Orthodontics Congress held in Maringá, a city inland of Brazil. Everyone was overjoyed with the power of quality and content disclosed by the presentations, and that was heard through the walls of the congress. One could certainly claim that was the best congress held in the last years. 

Many missed that opportunity; thus, the quality of the event could not be estimated by the number of attendees in the audience. The best indicator to value the event was the number of professionals moving around the areas where speeches were not given: the outer rooms and the trade fair were empty and reminded the streets of Brazilian cities when the national football team plays. Nobody dared to miss all the information being disclosed in those rooms. Even the social networks clamored for access.

In spite of all the themes being discussed, one in particular caught my attention: how much is it worth a smile? Based on the book '*O Valor da Beleza*' (The Value of Beauty),^1^ Dr. Carlos Câmara brilliantly discussed about a patient who, after being subjected to significant changes in smile esthetics, would earn about US$230,000 throughout his professional life due to this changes. Accurately determining how much a smile is worth it is not an easy task, and that was not Câmara's intention and neither is mine. The aim was not only to show how noble our job is, but also to cause those who dream about making the world a better place - by changing people's faces and smiles - to feel unease. 

The following question is paramount: If one single smile is worth so much, how much is it worth the knowledge necessary for an orthodontist to be able to change the lives of so many people? Millions; perhaps, billions of dollars. Patients' beautiful smiles and the satisfaction they feel as a result of the changes brought about by the orthodontist's work are indeed inestimable. The opposite, unfortunately, is also true: The disappointment and unhappiness caused by a bad deed can bury us in deserted lands. Undoubtedly, knowledge is a powerful tool that enables us to separate the wheat from the chaff. 

In Orthodontics, as in any other health specialty, knowledge can be acquired by reading scientific publications, attending scientific events and clinical meetings, and by being involved in daily clinical practice - splitted and all together. The idea will always be to share information, experience and skills. In order to do so, reading as well as critically and coherent thinking are treasures key to prevent one from running into serious trouble. 

Despite its economic value, there is something that goes beyond the power of money - which builds and ruins beauty -; something that goes beyond human existence and brings us near the pleasure of encountering the scientific true. Enormous pleasure derived from the possibility of improving someone else's life. Serving, improving and making others happier is priceless. Thus, why do some dental professionals refrain themselves from acknowledging the liberating and ludic power of knowledge, as described in the epigraph? There is a need to put lethargy aside and seek the oasis of our goal, as professionals and human beings, while crossing the desert.

In spite of the fact that humans are essentially conformed, we also hear: "- I feel confused with so much information." But as the writer and professor of Biochemistry Isaac Asimov would say: "If knowledge can create problems, it is not through ignorance that we can solve them." Scientific language is not simple and indeed needs change to reach a broader audience. Nevertheless, difficulty in understanding it cannot be used as a cane that supports ignorance. All the effort shall be rewarded financially, but above all, by a "thank you very much", an acknowledgement, a hand shake and/or a beautiful smile. 

All of sudden, you catch yourself recalling the smile of a child who wished to change the world.

David Normando - editor-in-chief (davidnormando@hotmail.com)
