# Supplementary material for: How much is it worth a smile?
Source: Dental Press J Orthod. 2015 May-Jun;20(3):11–2. doi: 10.1590/2176-9451.20.3.011-012.edt (PMC4520132; doi:10.1590/2176-9451.20.3.011-012.edt)
Supplement: Translation

# Translation: Quanto vale a beleza de um sorriso?

**Authors:** David Normando

O estudo em geral, a busca da verdade e da beleza são domínios em que nos é consentido permanecer crianças por toda a vida. **Albert Einstein (físico)**

Há bem pouco tempo, cerca de 400 ortodontistas participaram de Congresso de Ortodontia realizado em Maringá, uma cidade no interior do Brasil. O encantamento com a força da qualidade e do conteúdo das apresentações eram os sussurros que se ouviam das paredes do evento. Poder-se-ia dizer que foi o melhor congresso dos últimos. 

Muitos perderam essa oportunidade, por isso a qualidade do evento não poderia ser calculada pela quantidade de pessoas dentro do auditório. O melhor indicador para valorá-lo estava no número de profissionais circulando fora do ambiente das palestras: as salas externas e a área da feira comercial, todas vazias, lembravam as ruas do Brasil em dias de jogo da seleção de futebol. Dessa vez, ganhávamos de goleada. Ninguém queria perder qualquer informação que rolava dentro daquele ambiente. Até as redes sociais clamavam por acessos.

Não obstante os temas, chamou-me atenção um questionamento sobre o valor de um belo sorriso, ao estilo propaganda de creme dental. Baseado no livro *O Valor da Beleza* *1*, Carlos Câmara discorreu brilhantemente sobre uma pessoa que tivesse sofrido uma grande mudança em sua beleza, a qual ganharia, ao longo de sua vida profissional, cerca de 230 mil dólares advindos dessa mudança. Não é uma tarefa simples determinar, com exatidão, o custo de um sorriso. E essa não era a verdadeira intenção de Câmara, sequer é a minha aqui. O propósito era mostrar a nobreza do nosso trabalho e estimular a inquietação dos que sonham em tornar o mundo um lugar melhor, transformando rostos e sorrisos. 

A questão seguinte é fundamental: se a beleza de um único sorriso vale tanto, quanto valeria o conhecimento adquirido necessário para um ortodontista se habilitar a modificar a vida de tantas pessoas? Milhões, talvez bilhões. Um sorriso belo e a satisfação do seu paciente com a mudança provocada pelo seu trabalho são, de fato, incalculáveis. O oposto, infelizmente, é verdadeiro: a decepção e a tristeza causadas por uma má feitura também nos enterram nas areias do deserto. Não temos dúvida de que o conhecimento é uma poderosa ferramenta para separar o joio do trigo. 

Na Ortodontia, assim como em outras áreas da saúde, o conhecimento pode ser adquirido em leituras científicas, eventos, reuniões clínicas e no próprio exercício do cotidiano - tudo junto e misturado. A ideia será sempre a de compartilhar informações, experiências e traquejos. Para isso, entretanto, a leitura e o pensamento crítico e coerente são tesouros fundamentais para não embarcar em embrolhos. 

A despeito do seu valor econômico, há algo além da força da grana - que ergue e destrói coisas belas -, que transcende a existência humana e nos aproxima dos deleites da verdade científica. Prazer, sem medidas, por conta da possibilidade de melhorar a vida de outrem. Servir, aperfeiçoar e tornar "o outro" mais feliz é um trabalho impagável. Então, por que alguns colegas se enclausuram e não conseguem perceber a força libertadora, ao mesmo tempo lúdica, do conhecimento, como relatado na epígrafe desse texto? Faz-se necessário esquecer a letargia, e buscar, durante a travessia do deserto, o oásis de seu objetivo profissional e humanístico.

A despeito da acomodação da condição humana, também ouvimos: "- Eu me confundo com tanta informação." Se o conhecimento pode criar problemas, não é através da ignorância que podemos solucioná-los, escreveu o bioquímico e escritor Isaac Asimov. A linguagem científica não é simples e, de fato, precisa ser modificada para atingir um público mais amplo. Mas a dificuldade em compreendê-la não pode ser usada como bengala para apoiar a ignorância. Todo esforço será devidamente recompensado financeiramente e, principalmente, por um "muito obrigado", um reconhecimento, um aperto de mãos e/ou um belo sorriso. 

De repente, você voltará a se recordar, dentro de si, do sorriso daquela criança que queria mudar o mundo.

David Normando - editor-chefe (davidnormando@hotmail.com)
